# Elucidating the Nutritional Profile and Biochemical Characterization of High-Energy Nutritional Bar Formulated with Sukkari Date Paste and Mixed Nuts

**DOI:** 10.3390/foods14213661

**Published:** 2025-10-27

**Authors:** Hassan Barakat, Hani A. Alfheeaid, Thamer Aljutaily, Raed Alayouni, Hend F. Alharbi, Woroud A. Alsanei

**Affiliations:** 1 Department of Food Science and Human Nutrition, College of Agriculture and Food, Qassim University, Buraydah 51452, Saudi Arabia; h.alfheeaid@qu.edu.sa (H.A.A.); thamer.aljutaily@qu.edu.sa (T.A.); r.alayouni@qu.edu.sa (R.A.); hf.alharbi@qu.edu.sa (H.F.A.); 2 Department of Food and Nutrition, Faculty of Human Sciences and Design, King Abdulaziz University, Jeddah 21589, Saudi Arabia; walsanei@kau.edu.sa

**Keywords:** nutritional evaluation, high-energy bar, Sukkari Date, bioactive compounds, sustainable food systems, food supply

## Abstract

Growing health consciousness drives demand for convenient, nutrient-dense snacks. This study evaluates five Sukkari date-mixed-nut bar formulations (DNB1–DNB5; date/nut ratios 40:60–80:20) through comprehensive biochemical and nutritional analyses. Macronutrient profiling showed that higher date ratios increased moisture and carbohydrates, whereas higher nut ratios enhanced protein, fat, and caloric density. Mineral assays revealed progressive increases in calcium, phosphorus, magnesium, and trace elements as date content decreased. The assessment of phytochemicals and antioxidants demonstrated that total phenolics, flavonoids, and radical-scavenging activities peaked in nut-rich bars, declining by ~50% in date-rich bars, underscoring nuts’ dominant antioxidant role. HPLC profiling identified catechol and vanillic acid as the major phenolics, with optimal release and retention at the 60:40 ratio (DNB3). Amino acid (AA) analysis confirmed positive correlations between nut content and total/essential AAs; DNB1–DNB2 achieved favorable essential-to-nonessential AA ratios (0.56–0.59) and higher protein quality indices. Fatty acid (FA) composition analysis revealed that oleic acid was identified as the major constituent across all formulations, coupled with optimal omega-6/omega-3 ratios. GC-MS analysis identified a total of 31 volatiles, mainly benzene derivatives and FA methyl esters. Results also revealed that notable variations attributed to different date/nut ratios significantly alter aroma profiles, with DNB3 yielding the most remarkable diversity of health-associated volatiles. Results from PCA and hierarchical clustering suggest that a single dominant dimension (PC1, 94.47% variance) governs compositional differences among the five date bar formulations, reflecting deliberate variation in ingredient proportions. The evidence suggests that DNB3’s (60:40 Sukkari date to mixed nut ratio) delivers balanced macro-nutrients, robust antioxidants, and diverse bioactives, positioning it as a health-promoting functional snack, aligning with its suitability for athletes, clinical nutrition applications, and health-conscious populations. These findings support the commercial development of optimized date-nut bars as nutrient-dense functional snacks, and future work should focus on scale-up production, shelf-life stability, and assessing in vivo bioavailability.

## 1. Introduction

High-energy nutritional bars have emerged as convenient, nutrient-dense snack formats tailored to meet the heightened dietary demands of athletes, shift workers, and individuals with increased energy requirements [1]. By developing nutritionally balanced snack bars with immediate-acting carbohydrates, muscle-protective proteins, and energy-dense lipids, these bars serve to enhance long-term nutritional health benefits and effective performance [2]. The incorporation of age-old ingredients with cutting-edge processing techniques of these products has been instrumental in improving both their functional attributes and consumers’ acceptability [3]. In 2024, the global market for nutritional bars was valued at a remarkable growth, with an estimated value of approximately USD 7.4 billion and projected to reach USD 13.2 billion by 2034, with a notable compound annual growth rate of 6.1% [4]. This growth reflects rising health awareness and demand for convenient nutrition. Additionally, the functional food market is predicted to reach USD 441.66 billion by 2028, with nutritional bars involving protein, energy, meal-replacement, and specialty (e.g., gluten-free, organic, plant-based) formulations and constituting a key segment [3].

A recent study demonstrates that consumers are increasingly motivated by health benefits when selecting functional foods, with over 24.9% of male athletes preferring protein bars for quick energy and protein intake. This figure has doubled in three years [4]. Date fruits are recognized for their exceptional nutritional profile, comprising dietary fibers, natural sugars, micronutrients, and bioactive phytochemicals, including phenolic acids, polyphenols, and carotenoids that provide anti-inflammatory, antioxidant, and antimicrobial properties [5]. On the other hand, nuts are also a good source of high-quality proteins, essential fatty acids (FAs), vitamins, and minerals. This makes the date-nut combination a great base for nutrient-dense functional snack bars aimed at health-conscious customers [6].

Sukkari date (*Phoenix dactylifera* L.) is a premium cultivar widely grown in Saudi Arabia, prized for its soft texture, golden hue, and high natural sugar content [7,8]. Its carbohydrate nature, which is mostly glucose and fructose, makes it easy to get energy back after a meal quickly. Thus, the incorporation of Sukkari date paste into functional foods aligns with current trends toward a natural sweetener and binder for nutritious bar formulations [5,9]. Moreover, Sukkari dates contribute dietary fiber (4–8% *w*/*w*), which promotes gastrointestinal health and attenuates glycemic excursions [10]. Additionally, phenolic compounds in such dates significantly enhance their antioxidant capacity, thereby counteracting oxidative stress implicated in various chronic diseases [11,12,13].

Incorporation of mixed nuts like almonds, walnuts, and cashews to the mix increases the nutritious profile by adding high-quality plant proteins, unsaturated fatty acids, and a variety of micronutrients, including vitamin E, magnesium, and phosphorus [14]. Almonds and walnuts are especially high in monounsaturated and polyunsaturated fats, which are associated with decreasing the cardiovascular risk markers and improvement of blood lipid profiles [15,16]. Cashews and pistachios containing essential AAs and bioactive phytochemicals are related to enhancing the antioxidant and anti-inflammatory potential of different formulated bars [15,17]. This mix of date paste and mixed nuts has a lot of nutritional value, making it great for high-energy uses. Several studies have shown that date-based nutritious bars made with a variety of dietary items are good for your health and nutrition [5,9,10]. Similarly, Parn et al. [18] formulated novel fruit bars utilizing two popular date cultivars, including Sukkari, showing enhanced nutritional composition and consumer acceptability. Interestingly, mixed nuts provide unsaturated fats, plant proteins, and bioactive substances to the bars’ health advantages. Mixed nuts maintain steady postprandial blood glucose levels compared to processed snacks and improve gut microbiome composition by increasing *Bifidobacterium longum* and reducing inflammation-associated species. [19,20]. Nonetheless, these individual components have undergone substantial examination; their synergistic effects in bar compositions, especially with premium date cultivars like Sukkari, remain inadequately defined. Several studies have shown that nutritious bars made with dates are good for your health and nutrition. Hong et al. [21] found that mixed nut consumption significantly reduced cardiovascular disease risk factors, including decreased triglycerides, total cholesterol, and LDL-cholesterol, while increasing adiponectin and reducing oxidative stress biomarkers [22]. These qualities make date-nut bars useful for sustained energy, cardiovascular health promotion, and metabolic control, especially for athletes, health-conscious customers, and groups who need efficient nutrition delivery [23,24].

Despite the documented nutritional benefits of date-based bars and mixed nuts separately, a critical knowledge gap exists regarding the comprehensive characterization of Sukkari date-mixed nut bars specifically. While Parn et al. [18] discovered enhanced nutritional value in Sukkari date bars, despite the absence of mixed nuts or AAs and FAs profiles. The previously described date-nut studies have concentrated on different types of dates or limited nutritional variables. This makes it hard to see their bioactive compounds, whole amino acid profile, and mineral content for use in functional foods. Furthermore, no research has thoroughly investigated the antioxidant activity of Sukkari date-mixed nut bars in relation to diverse phenolic profiles. Such a knowledge gap restricts the potential for developing a functional food for populations such as athletes and health-conscious consumers, who require high-energy and nutrient-dense products. Therefore, this study aims to develop high-energy bars formulated with varying ratios of Sukkari date paste and mixed nuts, focusing on their nutritional composition and biochemical characteristics, including FA and AA profiles, mineral quantification, phenolic compound identification, and antioxidant activity assessment.

## 2. Materials and Methods

### 2.1. Raw Ingredients

All ingredients, such as Sukari date paste and Sukkari date powder, were purchased from Al Emtyaz Dates Factory, Buraydah, Qassim Region, SA (https://www.alemtyaz.com.sa, accessed on 24 March 2024). At the same time, nuts, spices, and cow’s milk powder were obtained from Damascus Winds supermarket, Buraydah, Qassim Region, SA. For each 1 kg nuts mix, dried Sukkari date powder (200 g), roasted almond (150 g), pistachio (100 g), whole oats (100 g), cow’s milk powder (100 g), roasted cashew (75 g), roasted sesame seeds (75 g), walnuts (50), hulled pumpkin seeds (50 g), hulled sunflower seeds (50), chia seeds (30), dried cinnamon powder (10 g), and dried ginger powder (10 g) were mixed in a knife blender (Santos, VM0122E, Cleveland, OH, USA) at speed 3 for 60 sec min to prepare a homogenous nut mix and then kept at 4 ± 1 °C before further processing.

### 2.2. Formulation of High-Energy DNB

**Figure 1:**
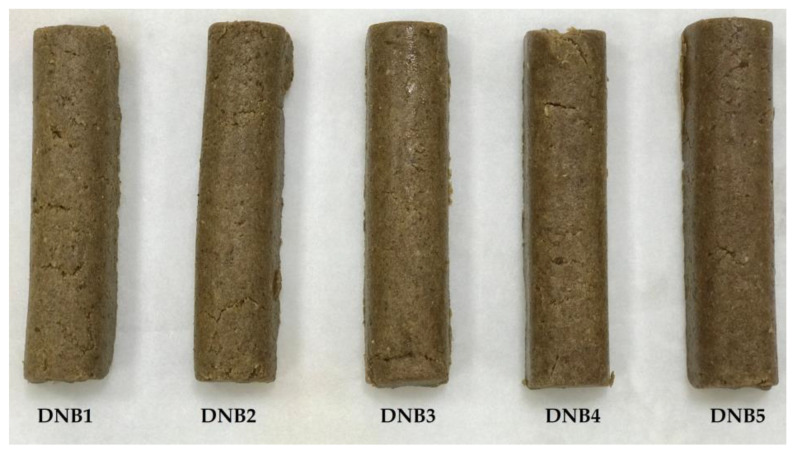
Formulas of high-energy DNB bars. The ingredients of formulas DNB1 to DNB5 were presented in Table 1.

For each formula, the prepared date paste was thoroughly mixed with the predetermined nut mix in accordance with the ratios specified in Table 1 to create a uniform dough. The dense and sticky nature of date paste ensures effective incorporation of nuts and improves the cohesiveness of the bar matrix. Each formula was mechanically mixed using a mixer until the mixture resembled a homogeneous consistency mix. Immediately after mixing, the date-nut mix was portioned using a ready shaping frame and pressed systematically to guarantee uniform compaction and consistent thickness throughout each bar. The shaped bars (35 each) were then wrapped with polyethylene sheets and subjected to storage under cooling conditions of 4 ± 1 °C until further analysis; see Figure 1.

### 2.3. Nutritional Composition and Mineral Content of Different DNBs

All DNB formulas underwent comprehensive chemical analyses to quantify moisture (AOAC 925.10), crude protein (AOAC 981.10), crude lipid (AOAC 963.15), ash (AOAC 942.05), dietary fiber (AOAC 992.16), available carbohydrate contents (by difference), and caloric value, following established AOAC procedures [25]. Mineral composition, specifically sodium and potassium levels, was assessed through flame photometry (Cambridge, UK) following AOAC 970.35. At the same time, atomic absorption spectroscopy was utilized to measure concentrations of calcium, magnesium, iron, copper, manganese, and zinc in accordance with AOAC protocols employed by a PerkinElmer AAnalyst™ 400 (Waltham, MA, USA) with appropriate hollow-cathode lamps and standard curves per AOAC 984.27 [25]. Furthermore, phosphorus content was determined using a standardized colorimetric method as outlined by Borah et al. [26], measuring absorbance at 880 nm using a UV–Vis spectrophotometer (Shimadzu UV-1800, Kyoto, Japan). All measurements were performed in triplicate, and calibration standards and quality controls were included in each analytical batch.

### 2.4. Phytochemical Analysis of Different DNBs

The total phenolic compounds (TPC) in DNBs were quantified using the Folin–Ciocalteu method, with results expressed as milligrams of gallic acid equivalents (mg GAE per 100 g), following the procedure outlined by Bettaieb et al. [27]. Total carotenoid (TC) content was measured colorimetrically according to a modified protocol [28]. Antioxidant capacity, assessed via DPPH radical scavenging activity (DPPH-RSA), involved a colorimetric assay using 2,2-diphenylpicrylhydrazyl (DPPH) radicals, calculating the percentage of radical inhibition and referencing a Trolox standard, with results reported as micromoles of Trolox equivalents per gram dry weight (μmol TE g^−1^) [29]. Radical scavenging activity against ABTS radicals (ABTS-RSA) was tested using the method described by [30]. A 0.1 mL aliquot of extract was combined with 2.9 mL of ABTS solution, which was generated by incubating 7 mmol L^−1^ ABTS and 2.45 mmol L^−1^ potassium persulfate in distilled water, in the dark at room temperature for 12–16 h. Before use, this solution was diluted with ethanol to an absorbance of 0.70 ± 0.02 at 734 nm and equilibrated at 30 °C. Sample aliquots were then added, incubated at 30 °C for 20 min, and the absorbance reduction at 734 nm was recorded using a UV-Vis spectrophotometer (Shimadzu UV-1800, Kyoto, Japan). Antioxidant capacity was calculated as μmol Trolox equivalents per gram of sample (μmol TE g^−1^) from a Trolox standard curve. Additionally, total flavonoid (TF) and total flavonol (TFL) contents were determined using methods by Barakat & Almundarij [31] and Kumaran & Karunakaran [32], respectively, with values expressed as milligrams of quercetin equivalents per gram (mg QE g^−1^).

### 2.5. Quantification of Phenolic Compounds of Different DNBs

According to Schneider [33], phenolic compounds in different DNBs were analyzed using an Agilent 1260 Infinity HPLC system (Agilent Technologies, Palo Alto, CA, USA) equipped with a quaternary pump, autosampler, and a variable wavelength detector (VWD, Hewlett Packard 1050) set at 280 nm. The chromatographic separation was performed on a Kinetex^®^ 1.7 µm EVO C18 column (150 mm × 4.6 mm, Phenomenex, Torrance, CA, USA) maintained at 30 °C. A ternary linear gradient elution was employed, consisting of (A) HPLC-grade water with 0.1% trifluoroacetic acid (TFA), (B) acetonitrile, and (C) HPLC-grade methanol. The gradient program was initiated at 94:3:3 (*v*/*v*) for phenolics and 98:1:1 (*v*/*v*) for flavonoids. Automated injection of 20 µL extract was used, with a flow rate of 1 mL min^−1^, column temperature of 30 °C, and ambient laboratory temperature of 20 °C. Quantification of phenolic peaks was carried out in mg kg^−1^ using external calibration curves of appropriate standard solutions.

### 2.6. Determination of AA Profile in Different DNBs

The AA composition of various KBs enriched with GMB was analyzed using a Sykam AA analyzer (Sykam GmbH, Eresing, Bavaria, Germany). The system was configured with a solvent delivery unit (S 2100 quaternary pump), an autosampler (S 5200), an AA reaction module (S 4300) equipped with a dual-filter photometer operating at 440–570 nm with constant signal output and signal integration capability, and a refrigerated reagent organizer (S 4130). The analysis was performed using an LCA K06/Na column under gradient elution. Buffer A was prepared with 11.8 g trisodium citrate dihydrate, 6 g citric acid, 65 mL methanol, 6.5 mL of 32% HCl, and 0.5 g phenol per liter (pH 3.45, normality 0.12). In comparison, buffer B contained 19.6 g trisodium citrate dihydrate, 3.1 g NaOH, and 5 g boric acid per liter (pH 10.85, normality 0.20). The system operated at a flow rate of 1 mL min^−1^ with a programmed column temperature gradient between 57 and 74 °C. Detection was monitored at wavelengths of 440 and 570 nm. A standard stock solution (Sykam Type H, CAT No. S000029) containing 18 AAs at 2.5 µmol mL^−1^ (cystine at 1.25 µmol mL^−1^) in 0.1 N HCl with 0.1% phenol was prepared. Standards were diluted (60 µL in 1.5 mL HPLC water), filtered (0.22 µm), and injected (100 µL). Samples (~1 g, weighed to four decimal places) underwent acid hydrolysis with 20 mL of 6N HCl at 110 °C for 16 h in sealed vessels. Hydrolysates were filtered, evaporated, reconstituted in 100 mL HPLC water, diluted 1:10, filtered, and injected (100 µL). AA concentrations were calculated using relative response factors from standards according to Cohen et al. [34]. Biological value and AA scores were determined following WHO guidelines (2007) [35] as described by Chavan et al. [36] using relevant equations.

### 2.7. Determination of the FA Profile in Different DNBs

According to Aldai et al. [37], the total FA components were converted into their corresponding methyl esters. These FA methyl esters (FAMEs), derived from the different date–nut bar (DNB) samples, were analyzed using gas–liquid chromatography (GLC) equipped with a flame ionization detector (FID). The chromatographic separation was performed under a programmed temperature gradient, starting at 100 °C and gradually increasing to 200 °C, with additional steps including a rise at 2 °C per minute until 230 °C, followed by a 10 min isothermal hold. The injection and detector ports were maintained at 250 °C and 300 °C, respectively. Data acquisition and peak integration were carried out using the Saturn GC Workstation Software, version 5.51.

### 2.8. Quantification of Volatile Components in Different DNBs

The chemical composition of NDB was performed using a Trace GC-TSQ mass spectrometer (Thermo Scientific, Austin, TX, USA) with a direct capillary column TG–5MS (30 m × 0.25 mm × 0.25 µm film thickness). The column oven temperature was initially held at 50 °C and then increased by 5 °C /min to 250 °C, held for 2 min, and then increased to the final temperature of 300 °C by 30 °C /min and held for 2 min. The injector and MS transfer line temperatures were kept at 270 and 260 °C, respectively; helium was used as a carrier gas at a constant flow rate of 1 mL/min. The solvent delay was 4 min, and diluted samples of 1 µL were injected automatically using Autosampler AS1300 coupled with GC in the split mode. EI mass spectra were collected at 70 eV ionization voltages over the range of *m*/*z* 50–650 in full scan mode. The ion source temperature was set at 200 °C. The components were identified by comparison of their mass spectra with those of the WILEY 09 and NIST 14 mass spectral databases.

#### Methylation Method

H_2_SO_4_-methanol 2% (*v*/*v*) was added to a vial containing 10 mg of the sample that had previously been weighed. The vial was heated at 80 °C with occasional shaking. Afterwards, 0.25 mL of the neutralized aqueous solution (sodium hydroxide 1 M) was added, and it was smoothly shaken. Allowed to cool at room temperature, then after neutralization, 1 mL KCl 59.64 g L^−1^ was added and shaken vigorously, then 1 mL hexane was added and shaken vigorously, then the upper organic layer was taken for GC-MS analysis following the methodology of Hewavitharana et al. [38].

### 2.9. Statistical Analysis

Statistical evaluation was performed using one-way ANOVA in SPSS version 22 (IBM Corp., Armonk, NY, USA, 2013) under a completely randomized design. Post hoc multiple comparisons were conducted using Duncan’s test at a *p* < 0.05 significance level according to Steele et al. [39]; all analyses were performed in triplicate. Additionally, principal component analysis (PCA) was performed to identify underlying patterns and relationships within the dataset. The PCA was performed on the 5 date bar samples (DNB1-DNB5) across 27 nutritional and bioactive variables. The data were standardized using z-score normalization before analysis to account for different scales and units across variables.

## 3. Results

### 3.1. Nutritional Composition of Formulated High-Energy DNBs

The nutritional composition of the five Sukkari DNBs (DNB1–DNB5) varied significantly (*p* < 0.05) with changing date-to-nut ratios (Table 2). Moisture content rose considerably from 9.68% in DNB1 to 13.43% in DNB5, reflecting increased date paste (DNB1 vs. DNB5, *p* < 0.05). Conversely, protein significantly declined from 10.89% to 5.567% (DNB1 vs. DNB5, *p* < 0.05), and total fat significantly decreased from 18.66% to 6.62% (*p* < 0.05). Ash content showed a modest but significant reduction (3.16% to 2.92%; DNB1 vs. DNB5, *p* < 0.05). Dietary fiber remained relatively constant (5.74–6.23%; *p* > 0.05). Total carbohydrates significantly increased from 57.62% in DNB1 to 71.47% in DNB5 (*p* < 0.05), while energy value decreased significantly from 417.01 kcal in DNB1 to 344.74 kcal in DNB5 (*p* < 0.05).

### 3.2. Mineral Content of Formulated High-Energy DNBs

The mineral analysis of the five formulated bars (DNB1–DNB5) revealed significant differences (*p* < 0.05) across all measured elements with changing date-to-nut ratios (Table 3). Calcium content showed the most pronounced variation, decreasing from 422.12 mg 100 g^−1^ in DNB1 to 241.06 mg 100 g^−1^ in DNB5, with statistically significant differences between all formulations (*p* < 0.05). Potassium exhibited a similar declining trend from 1247.08 mg 100 g^−1^ (DNB1) to 1079.54 mg 100 g^−1^ (DNB5), though differences between DNB2, DNB3, and DNB4 were not significant (*p* > 0.05). Phosphorus content decreased substantially from 785.95 to 430.77 mg 100 g^−1^ across the formulation range, with significant differences between most treatments. Magnesium followed a similar pattern, declining from 326.52 mg 100 g^−1^ (DNB1) to 195.66 mg 100 g^−1^ (DNB5), while sodium decreased from 57.54 to 35.97 mg 100 g^−1^. Among trace elements, manganese showed significant reductions from 3.57 mg 100 g^−1^ (DNB1) to 1.95 mg 100 g^−1^ (DNB5), with all formulations differing significantly (*p* < 0.05). Copper content declined from 1.67 to 0.91 mg 100 g^−1^, though some intermediate formulations showed non-significant differences. Iron decreased from 6.97 to 4.02 mg 100 g^−1^, with DNB4 and DNB5 showing similar values (*p* > 0.05). Zinc content reduced from 5.46 mg 100 g^−1^ (DNB1) to 2.91 mg 100 g^−1^ (DNB5), while selenium decreased from 21.89 µ100 g^−1^ to 11.84 µ100 g^−1^, with significant differences between most formulations (*p* > 0.05).

### 3.3. Antioxidant Activities of Formulated High-Energy DNBs

The antioxidant activity and phytochemical content of the five formulated high-energy bars (DNB1–DNB5) demonstrated significant variations (*p* < 0.05) corresponding to different date-to-nut ratios (Table 4). Total phenolic content (TPC) decreased progressively from 37.98 mg GAE g^−1^ in DNB1 to 18.17 mg GAE g^−1^ in DNB5, with all formulations showing statistically significant differences (*p* < 0.05). DPPH radical scavenging activity (DPPH-RSA) followed a similar declining trend, ranging from 48.29 μmol TE g^−1^ in DNB1 to 23.09 μmol TE g^−1^ in DNB5, with significant differences between all treatments. ABTS radical scavenging activity (ABTS-RSA) exhibited the highest values in DNB1 (62.76 μmol TE g^−1^) and progressively decreased to 27.71 μmol TE g^−1^ in DNB5, with statistical significance across all formulations (*p* < 0.05). Total flavonoids (TF) content declined from 26.01 mg QE g^−1^ (DNB1) to 10.91 mg QE g^−1^ (DNB5). In comparison, total flavonols (TFL) decreased from 20.80 mg QE g^−1^ to 7.75 mg QE g^−1^, both showing significant differences between most formulations (*p* < 0.05).

### 3.4. Phenolic Profile of Formulated High-Energy DNBs

The HPLC analysis of phenolic acids and flavonoids in formulated Sukkari bars (Table 5) revealed that catechol was the predominant phenolic acid across all formulations, with concentrations of 4183.11, 4640.26, and 3676.47 mg kg^−1^ in DNB1, DNB3, and DNB5, respectively. Vanillic acid was the second most abundant, peaking at 528.95 mg kg^−1^ in DNB3 and decreasing to 418.57 mg kg^−1^ in DNB5, indicating that a 60:40 date-to-nut ratio optimizes retention of this compound. Gallic acid and *p*-hydroxybenzoic acid also followed a similar pattern, with the highest levels in DNB3 (15.56 and 89.09 mg kg^−1^, respectively). Phenolic acids such as syringic and *p*-coumaric acids were found only in DNB3 (5.31 mg kg^−1^) and at trace levels in DNB1 and DNB5. Flavonoid profiling revealed quercetin as the major flavonol, with a concentration of 2.79 mg kg^−1^ in DNB1, 0.66 mg kg^−1^ in DNB3, and increased to 5.82 mg kg^−1^ in DNB5. Apigenin and rutin were the most abundant in DNB1 (1.48 and 2.11 mg kg^−1^, respectively) but declined with increasing date ratio, indicating that nuts contribute significantly to these compounds. Kaempferol and myricetin were detected at low levels (<0.6 mg kg^−1^) across all treatments. Overall, the 60:40 date-to-nut formulation (DNB3) accomplished the highest total phenolic acids (≈5282 mg kg^−1^) and substantial flavonoid levels, balancing date-derived antioxidants and nut-derived flavonols.

### 3.5. AA Composition of Different DNBs

High-energy DNB formulas were analyzed for their contents of AAs, and the data are shown in Table 6**.** The AAs analysis of the five formulated Sukkari-DNBs (DNB1–DNB5) revealed substantial compositional differences corresponding to varying date-to-nut ratios. The total amount of AAs was down from 8.47 g 100 g^−1^ in DNB1 to 5.29 g 100 g^−1^ in DNB5. The amounts for DNB2, DNB3, and DNB4 were 7.99, 8.02, and 6.26 g 100 g^−1^, respectively. Leucine was always the most common EAAs in all formulations, with amounts ranging from 0.33 g 100 g^−1^ in DNB5 to 0.57 g 100 g^−1^ in DNB1. Valine came in third, with levels ranging from 0.30 to 0.43 g 100 g^−1^, while phenylalanine came in second, with levels ranging from 0.31 to 0.46 g 100 g^−1^. There was a big difference in the amount of lysine, an important amino acid for nutrition, from 0.19 g 100 g^−1^ in DNB5 to 0.33 g 100 g^−1^ in DNB1. Methionine had the lowest levels of EAAs, with amounts ranging from 0.09 g 100 g^−1^ (DNB4) to 0.14 g 100 g^−1^ (DNB1). The total amount of EAAs went down from 3.03 g 100 g^−1^ in DNB1 to 1.97 g 100 g^−1^ in DNB5. In all formulations, glutamic acid was the most common NEAAs, with the highest content in DNB1 (1.92 g 100 g^−1^) and the lowest in DNB5 (1.08 g 100 g^−1^). The second most common NEAA was aspartic acid, which ranged from 0.504 g 100 g^−1^ (DNB5) to 0.84 g 100 g^−1^ (DNB1). Proline levels ranged from 0.42 g 100 g^−1^ (DNB4) to 0.69 g 100 g^−1^ (DNB1). Arginine, on the other hand, had an opposite effect on nut content, peaking at 0.71 g 100 g^−1^ in DNB2 and dropping to 0.34 g 100 g^−1^ in DNB5. The total amount of NEAAs varied from 3.32 g 100 g^−1^ (DNB5) to 5.45 g 100 g^−1^ (DNB1). The EAAs/NEAAs ratios, on the other hand, ranged from 0.46 for DNB3 to 0.59 for DNB5. DNB1, DNB2, and DNB4 had intermediate values of 0.56, 0.59, and 0.56, respectively. These ratios reflect the changing protein quality characteristics across different formulations.

Table 7 displays the AAs%, computed biological efficiency, essential AA index (EAAI), and requirement index of different prepared bars. The five DNB bars exhibited apparent formulation-dependent differences in protein quality metrics. DNB1 had the most total AAs (8.48 g 100 g^−1^) and total essential AAs (3.03 g 100 g^−1^). DNB5 had the least (5.29 and 1.97 g 100 g^−1^, respectively). The highest levels of branched-chain AAs were in DNB2, where they reached 152.71 mg g^−1^ protein. The highest levels of basic AAs were in the same formulation, where they reached 168.73 mg g^−1^ protein. The biological value (BV) went from 43.06 (DNB1) to 52.05 (DNB5), with DNB2 at 50.44. The EAAI went from 53.90 (DNB4) to 56.27 (DNB2). DNB3 had the most total conditional AAs (526.57), while DNB4 had the least (462.32). Age-specific requirement indices for infants, preschoolers, schoolchildren, and adults exceeded 100 for all formulations, with DNB2 consistently showing the highest indices (120.55–150.67), indicating superior capacity to meet age-dependent AA needs.

### 3.6. FA Composition of Formulated High-Energy DNBs

The FA composition analysis revealed distinct patterns across the three selected DNB formulations (Table 8). Oleic acid (C18:1 n9) was the predominant FA, decreasing from 44.94% in DNB1 to 40.51% in DNB5, while palmitic acid (C16:0) increased substantially from 15.06% to 20.26%. Total saturated FAs increased progressively from 24.05% in DNB1 to 32.92% in DNB5, accompanied by corresponding decreases in monounsaturated FAs (45.63% to 41.38%) and polyunsaturated FAs (30.31% to 25.70%). Linoleic acid (C18:2 n6) content decreased from 27.38% in DNB1 to 23.12% in DNB5, while α-linolenic acid (C18:3 n3) remained relatively stable (2.90–2.52%), resulting in omega-6 to omega-3 ratios ranging from 9.0:1 to 9.5:1 across formulations.

### 3.7. GC-MS Volatile Profile Analysis of Formulated High-Energy DNBs

The GC-MS analysis of the three selected formulated Sukkari DNBs (DNB1, DNB3, and DNB5) with varying ratios of Sukkari date paste and nut mixture (40:60%) in DNB1 and (80–20%) in DNB5 revealed a complex volatile organic compound (VOC) profile comprising 31 distinct compounds (Table 9). The identified compounds belonged to several chemical classes, with benzene derivatives, FA methyl esters, and organic acids being the predominant groups. For benzene derivatives and aromatic compounds, the most significant findings were the detection of numerous benzene derivatives, which constituted the major portion of the volatile profile. Compounds such as benzene (1-butylhexyl)-, benzene (1-propylheptyl)-, benzene (1-ethyldecyl)-, and benzene (1-methylnonyl)- were identified across all formulations. These compounds are typically generated during thermal processing of nuts and thus closely correlate with the distinctive nutty and roasted flavors in nut-based food products.

Several FA methyl esters were detected, including 7-hexadecenoic acid methyl ester and 17-octadecenoic acid, which are essential volatile compounds in nuts and dates. The 7-hexadecenoic acid methyl ester, identified in peaks 19 and 22, is a monounsaturated FA derivatives that contributes to the characteristic aroma profile of date-based products. The analysis revealed the presence of various organic acids and their derivatives, including hexadecanoic acid (palmitic acid) and 9,12-octadecadienoic acid compounds. Hexadecanoic acid (peak 21, 26) is commonly found in nuts and dates and contributes to the fatty, waxy notes in the aroma profile. Notably, the volatile profile showed significant variation among the five formulations. DNB3 (60% date paste, 40% nut mixture) exhibited unique characteristics with several compounds, as shown in Table 9.

**Figure 2:**
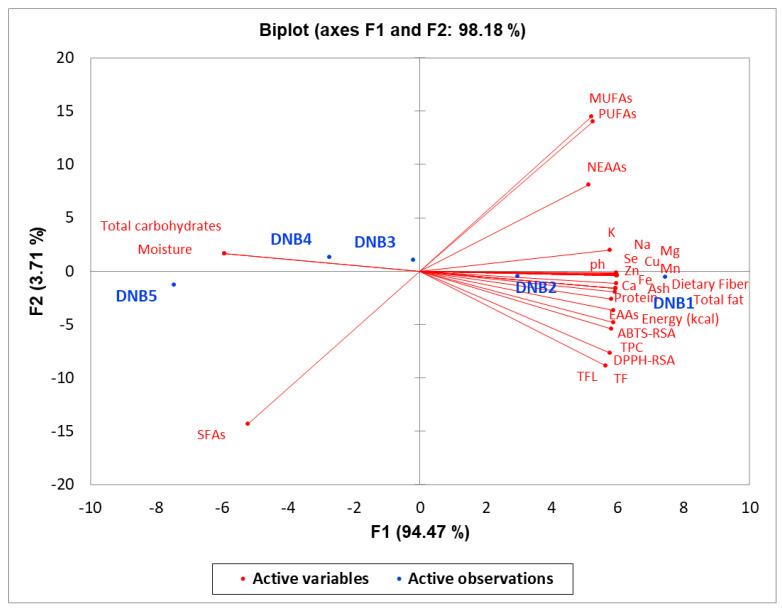
PCA biplot showing the relationships between samples and variables in the first two principal components.

**Figure 3:**
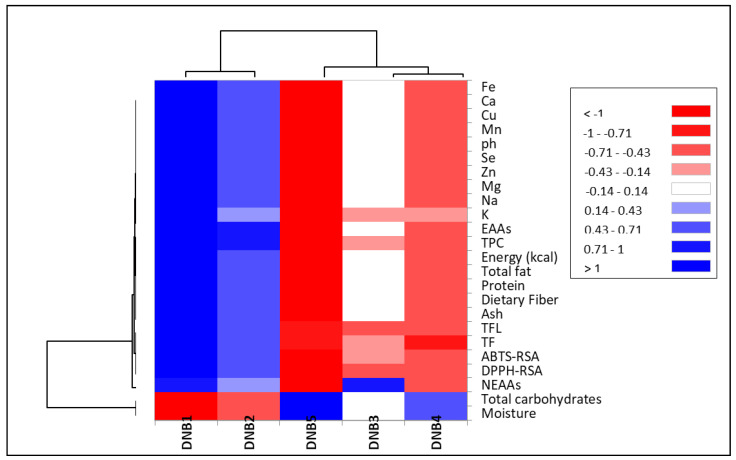
Heatmap with hierarchical clustering showing standardized values of nutritional and bioactive components across five date bar formulations.

Interestingly, the principal component analysis (PCA) of all measured variables is clearly presented for DNB1–DNB5 in Figure 2. The first two principal components explained 98.18% of total variance, with PC1 accounting for 94.47% and PC2 for 3.71%, indicating a dominant pattern of systematic variation among formulations. Samples showed clear separation along PC1 in a gradient pattern from DNB1 to DNB5. Hierarchical clustering grouped DNB1-DNB2 as nutrient-dense formulations and DNB4-DNB5 as lower nutrient-density samples (Figure 3). Minerals (Se, Zn, P, Mn, Ca, Cu, Fe, Mg, Na), macronutrients (protein, total fat, energy, dietary fiber), and bioactive compounds (TPC, DPPH-RSA, ABTS-RSA, TF, TFL) showed strong positive associations with PC1. In contrast, moisture and total carbohydrates were negatively associated. EAAs, MUFAs, and PUFAs also contributed positively to PC1. The heatmap with hierarchical clustering revealed tight co-variation among minerals and antioxidant compounds, indicating coherent nutritional patterns across formulations. The systematic gradient reflects a compositional trade-off between nutrient density and straightforward carbohydrate content, with DNB1 representing the most nutrient-dense formulation (Figure 3).

## 4. Discussion

The global nutritional bar market is undergoing rapid expansion, driven by growing health awareness and consumer demand for convenient and nutrient-dense snacks. Fruit-based snack bars are the healthiest, with natural sugars, vitamins, minerals, and other bio-nutrients to fulfill consumers’ daily nutritional needs [40]. Date-based bars have materialized as a promising subcategory. The date palm market is predicted to grow, and the global nuts market, supplying essential proteins and healthy fats, is estimated to rise. Date fruits had anti-inflammatory, anti-tumor, antihypertensive, anti-hypercholesterolemia, and antimicrobial properties [41,42,43]. Sukkari dates contribute dietary fiber, natural sugars, micronutrients, and bioactive compounds (phenolic acids, polyphenols, and carotenoids) associated with anti-inflammatory, antioxidant, and antimicrobial effects [5]. Mixed nuts provide high-quality proteins, essential FAs, vitamins, and minerals [44]. Together, these ingredients form a synergistic matrix ideally suited for developing functional, high-nutrition snack bars that fulfill modern consumer preferences for healthful, on-the-go foods.

The inverse relationship between date content and both protein and fat levels reflects the low protein (2.5–4.7%) and fat (<1%) of Sukkari dates compared to nuts, which contribute 15–25% protein and 40–60% fat [44,45]. DNB1 (60% nuts) demonstrated the highest protein and lipid content, supporting its capability as a protein-rich, energy-dense snack [46]. In contrast, DNB5 (80% dates) presented higher moisture and carbohydrate contents, delivering natural sugars and moisture maintenance characteristic of date-based formulations [47]. The consistent dietary fiber across formulations (≈6%) highlights the complementary contributions of date pulp and oat/nut matrix to fiber content [48]. The significant increase in total carbohydrates and corresponding decrease in caloric density with higher date ratios align with the dilution of lipid energy and highlight the trade-off between quick-release carbohydrates and sustained energy from fats [49,50]. Ash content reduction suggests minor mineral dilution by carbohydrate-rich dates, although all formulations remain good mineral sources. Statistically, each nutritional parameter differed significantly across formulations (*p* < 0.05), except dietary fiber, indicating that formulation adjustments can precisely tailor macronutrient profiles to nutritional targets. Ultimately, DNB1–DNB3 balance protein, fat, and energy optimally for sports and meal-replacement applications, while DNB4–DNB5 prioritize carbohydrate energy and palatability for daytime snacking.

The decline in mineral content with increasing date proportion reflects the fundamental compositional differences between dates and nuts, where the mineral profile of blended nuts surpasses that of date paste [44,51]. The substantial calcium reduction (43% from DNB1 to DNB5) aligns with reported values for nuts, particularly almonds and pistachios, compared to date fruits [52,53]. This trend has significant dietary implications, as calcium deficiency is prevalent globally, and nut-rich formulations could contribute substantially to daily calcium requirements [54]. The trend of declining potassium content across formulations; however, its overall high concentration remained markedly, reflecting the natural mineral profile of the constituents of both dates and nuts [14,47]. These levels support cardiovascular health and blood pressure regulation, particularly relevant for active populations and individuals with hypertension risk [40]. The phosphorus reduction (45% decrease) underscores nuts’ superior phosphorus density, with implications for energy metabolism, bone health, and cellular function [14]. Magnesium content patterns reflect the remarkable magnesium density of nuts compared to dates, with DNB1 providing levels that could significantly contribute to daily magnesium requirements [53,55]. Magnesium deficiency is associated with metabolic disorders, muscle dysfunction, and cardiovascular disease; therefore, the increased magnesium content observed in these formulations offers notable health advantages [56]. Magnesium protects against oxidative stress by stabilizing and activating antioxidant enzymes (e.g., SOD, catalase, glutathione reductase) and supporting glutathione synthesis and mitochondrial function, thus reducing reactive oxygen species and lipid peroxidation [57].

The trace element profiles demonstrate nuts’ superiority as sources of bioavailable minerals necessary for enzymatic functions and antioxidant defense systems. The zinc content reduction from DNB1 to DNB5 reflects the high zinc density in nuts compared to dates, with associations for immune function, wound healing, and protein synthesis [14,55]. Similarly, copper and manganese reductions align with nuts’ excellent trace element profiles, where these minerals serve as cofactors for antioxidant enzymes and metabolic processes [53]. Iron content, while declining with increased date proportion, remained significant across all formulations, contributing to iron intake requirements, particularly important for women and athletes [58]. The selenium content, though reduced in higher date formulations, remained within ranges that could support antioxidant defense systems and thyroid function [59]. The statistical analysis reveals that formulation adjustments can precisely control mineral profiles, with most minerals showing significant differences between extreme formulations (DNB1 vs. DNB5). The non-significant differences between some intermediate formulations (DNB2, DNB3, DNB4) for certain minerals suggest gradual transitions that allow fine-tuning of mineral content to meet specific nutritional objectives. The high mineral density inherent in DNB1 and DNB2 provides a suitable platform for development into functional foods aimed at improving mineral status in at-risk populations and athletes [9]. The mineral profiles support the bars’ potential as convenient sources of essential minerals often inadequate in modern diets, particularly calcium, magnesium, and trace elements [54,56]. Higher date formulas (DNB4, DNB5), while providing lower mineral density, offer adequate mineral content combined with natural sugars, fiber, and bioactive compounds characteristic of dates, making them suitable for general snacking applications where mineral density is less critical than palatability and energy provision [40].

The fact that the antioxidant capacity and phytochemical content go down as the date proportion goes up shows that mixed nuts are better at fighting free radicals than date paste, which goes against what you might think based on dates’ reputation as antioxidant-rich fruits [5,47]. The large drop in TPC (52% from DNB1 to DNB5) is in keeping with what has been said about the phenolic content of nuts, especially walnuts, almonds, and pistachios, when compared to fresh dates [14,60]. This finding suggests that phenolic compounds in the nut mix, including ellagic acid, gallic acid, and catechins derived from almonds and walnuts, are instrumental in enhancing the bars’ oxidative defense properties [44]. The DPPH and ABTS radical scavenging activities were substantially linked to the TPC values. This is in line with other research that found a favorable link between phenolic content and antioxidant capacity in nut-based products [61,62]. The higher ABTS-RSA results than DPPH-RSA values for all formulations show that ABTS assays can find a wider range of antioxidants since they evaluate both hydrophilic and hydrophobic antioxidant activity. DPPH, on the other hand, mostly finds hydrophobic antioxidants [62]. This distinction is especially significant in nut-based formulations that incorporate lipophilic antioxidants such as tocopherols and tocotrienols [14].

The flavonoid and flavonol content patterns reinforce the nuts’ contribution to phytochemical density, with compounds such as quercetin, catechins, and proanthocyanidins being abundant in nuts [44]. The 62% reduction in TF content from DNB1 to DNB5 indicates that nuts serve as primary flavonoid sources in these formulations, contributing compounds with established cardiovascular and neuroprotective benefits [14]. While dates contain flavonoids, including quercetin, rutin, and luteolin, their concentrations (1.74–3.39 mg catechin equivalent 100 g^−1^) are substantially lower than those found in nuts [60,61]. The enhanced antioxidant activity in DNB1 and DNB2 formulations highlights their potential role as functional foods for managing oxidative stress-related conditions, particularly among athletes and health-conscious populations [63]. The phenolic compounds identified in similar date-nut products, including gallic acid, ferulic acid, and catechins, have demonstrated anti-inflammatory, cardioprotective, and neuroprotective effects in clinical studies [5,61].

The declining antioxidant trends with increased date content reflect the trade-off between palatability and functional properties, where higher date formulations (DNB4, DNB5) prioritize natural sweetness and energy density over antioxidant capacity [46]. However, even the lowest values (DNB5) maintain meaningful antioxidant activity comparable to conventional snack bars, suggesting adequate functional benefits across all formulations [64].

The phenolic and flavonoid profiles of the formulated Sukkari date–nut bars underscore the intricate interplay between date pulp and nut matrices in determining antioxidant composition and potential bioactivity. Catechol emerged as the most abundant phenolic, reflecting the high catechol derivative content inherent to Sukkari dates and corroborating previous reports on the dominant role of catechol in date fruit antioxidant potential [47]. The peak catechol and vanillic acid levels observed in DNB3 (60% date) suggest that moderate date incorporation enhances phenolic extractability, likely due to optimal matrix porosity facilitating solvent penetration and phenolic release [65]. Results indicate that increased date content in DNB3 correlates with higher vanillic acid concentrations, a potent radical scavenger, compared to DNB5 (80% date), leading to phenolic-cell wall interactions, thus limiting phenolic bioavailability [49]. Gallic and *p*-hydroxybenzoic acids followed similar trends, reinforcing the notion that phenolic recovery is maximized at intermediate pulp ratios. The restricted detection of syringic and *p*-coumaric acids primarily in DNB3 further highlights the importance of formulation balance: these compounds, which possess anti-inflammatory and cardioprotective effects, were virtually absent in DNB1 and DNB5 formulations, suggesting that a 60:40 date-to-nut ratio fosters a synergistic matrix environment conducive to their liberation [10]. The overall total phenolic acid content peaked in DNB3, outperforming both DNB1 and DNB5 and underscoring the critical role of matrix structure in phenolic bioaccessibility [66].

Flavonoid analysis revealed quercetin as the principal flavonol, with its highest level in DNB5 aligning with reports that date peels are a rich quercetin source [66,67]. Conversely, apigenin and rutin peaked in DNB1, indicating that the nut mixture—composed of almond, pistachio, and other nuts—contributes substantially to these compounds, consistent with the high rutin content reported in almond and pistachio [68,69]. During extraction, a reduction in apigenin and rutin levels with increasing date ratio suggests competitive interactions between date phenolics and nut flavonoids, where increased date sugar concentrations potentially influence solvent polarity and flavonoid solubility [70]. Kaempferol and myricetin remained minor constituents across all formulations, in agreement with their low prevalence in both date and nut matrices [71].

From a functional perspective, the rich ensemble of phenolic acids and flavonoids confers robust antioxidant, anti-inflammatory, and cardioprotective properties to the date–nut bars. Catechol and gallic acid exhibit strong hydrogen-donating capacity, mitigating oxidative stress implicated in chronic diseases [47], while vanillic and *p*-coumaric acids have demonstrated endothelial-protective and anti-hyperglycemic effects in vivo [49,72]. Quercetin, rutin, and apigenin synergistically modulate inflammatory pathways and inhibit platelet aggregation, thereby promoting cardiovascular health in nut-enriched diets [69,73]. The DNB3 achieves an optimal balance, delivering high phenolic acid content alongside substantial flavonoid levels, thus maximizing the bar’s functional potential without overemphasizing either date or nut contributions. The phenolic acid and flavonoid profiles of Sukkari date–nut bars are highly formulation-dependent, with moderate date incorporation (60%) fostering superior release and retention of key bioactives.

The AA profile of the formulated Sukkari date-nut bars demonstrates the significant impact of ingredient ratios on protein quality and nutritional value. The progressive decline in total AA content with increasing date proportion directly reflects the fundamental difference in protein content between dates and nuts, confirming previous reports on the protein-diluting effect of fruit-based ingredients in mixed formulations [44,45]. Concerning protein quality assessment and EAA adequacy, the dominance of leucine across all formulations aligns with the typical AA pattern in date-nut products, where leucine represents approximately 10-12% of total protein content [46,48]. This abundance is nutritionally significant, as leucine serves as a key regulator of muscle protein synthesis and metabolic processes [74]. The high lysine content in nut-rich formulations (DNB1, DNB2) effectively compensates a critical limitation of lysine in plant-based dietary proteins, which often serve as the first limiting AAs [75]. When evaluated against the FAO/WHO/UNU [35] AA scoring pattern, DNB1 demonstrated superior protein quality potential. The leucine content (0.57 g 100 g^−1^) substantially exceeds the recommended 70 mg g^−1^ protein for preschool children, while lysine levels (0.334 g 100 g^−1^) approach the 58 mg g^−1^ protein requirement (FAO/WHO/UNU, 2007). However, the methionine + cysteine combination (0.361 g 100 g^−1^ total) remains potentially limiting relative to the 25 mg g^−1^ protein recommendation, consistent with typical sulfur AA limitations in plant-based proteins [76].

Regarding EAAs/NEAAs ratios and nutritional implications, the EAAs/NEAAs ratios approach the ideal value of 0.6 recommended for high-quality proteins, with DNB2 and DNB5 achieving ratios closest to this target (FAO/WHO/UNU, 2007). Interestingly, the higher ratios in date-rich formulations (DNB4, DNB5) result from proportionally greater reductions in NEAAs rather than EAAs preservation, suggesting differential AA stability during processing or inherent compositional differences between date and nut proteins [77]. For functionality, high glutamic acid content across all formulations plays a dual role in flavor enhancement via umami taste and potential health promotion [5]. The substantial arginine levels, particularly in DNB2, provide cardiovascular benefits through nitric oxide pathway enhancement. At the same time, the balanced essential AA profile supports various physiological functions beyond basic protein synthesis requirements [45]. Also, the AA profile reflects not only ingredient composition but also potential matrix interactions that may influence protein digestibility and bioavailability. The fiber-rich date matrix may create physical barriers affecting protein accessibility. At the same time, the lipid content from nuts could enhance the absorption of fat-soluble vitamins and modify protein digestion kinetics [46]. These factors suggest that AA bioavailability may vary among formulations independent of total content.

Based on comprehensive AA analysis, DNB1 emerges as the optimal formulation for protein quality, offering the highest total AA content, a superior EAAs profile, and a favorable EAAs/NEAAs ratio. This formulation successfully balances the nutritional benefits of dates—including natural sugars, fiber, and bioactive compounds—with the superior protein quality characteristics of mixed nuts [77]. DNB2 represents a viable alternative for applications requiring moderate protein content while maintaining sensory acceptability. In contrast, formulations with higher date content (DNB4, DNB5) are better suited for applications prioritizing carbohydrate energy and natural sweetness over protein density. The results support the feasibility of developing personalized nutritional products with flexible ingredient ratios to meet specific individual dietary and preference parameters [44].

The protein quality profiles demonstrate that a balanced date-to-nuts ratio enhances AAs adequacy and utilization efficiency in functional snack bars. DNB2’s high BV and EAAI reflect optimal alignment with FAO/WHO reference patterns for essential AAs. At the same time, its superior requirement indices across all age groups underscore its potential to support growth and maintenance (FAO/WHO/UNU, 2007). DNB3’s elevated conditional AAs suggest enhanced protein utilization under increased physiological demands, although its BV and EAAI are slightly lower. Overall, DNB2 emerges as the best formulation for maximizing protein quality, combining the natural sweetness, fiber, and bioactives of dates with the superior AA profile of mixed nuts to deliver a nutritionally robust snack suitable for diverse age groups [78,79].

The FA profiles demonstrate that DNB1 provides the most favorable nutritional composition from a cardiovascular health perspective, featuring the highest oleic acid content and the lowest saturated FA proportion. The predominance of oleic acid across all formulations aligns with Mediterranean diet principles. It supports cardiovascular benefits, as oleic acid consumption has been linked to improved HDL/LDL cholesterol ratios and reduced inflammatory markers [80]. The progressive increase in saturated FAs, particularly palmitic and myristic acids, from DNB1 to DNB5 may lessen the overall cardioprotective potential of higher-nut formulations, as excessive palmitic acid intake has been associated with increased cardiovascular disease risk compared to oleic acid-rich diets [81]. While the omega-6 to omega-3 ratios remain elevated compared to optimal recommendations (1–4:1), they fall within acceptable ranges for functional foods and reflect the nutritional limitations of plant-based ingredients in providing long-chain omega-3 FAs.

The GC-MS analysis of Sukkari date-nut bars revealed 31 volatiles whose profiles varied markedly with date-to-nut ratios. Benzene derivatives, originating from roasted nuts [82], dominated the aroma and imparted characteristic nutty notes. At the same time, FA methyl esters (e.g., 7-hexadecenoic and octadecenoic acid esters) reflected lipid breakdown and contributed fruity, sweet nuances [82,83]. Formulation DNB3 (60% date, 40% nuts) exhibited notably lower levels of several volatiles, indicating that ingredient ratio strongly influences volatile release and retention [46,84]. These compounds not only define sensory quality but also signal the presence of bioactive FAs and antioxidants inherent to dates and nuts, underscoring the bars’ nutritional value [71,83]. The consistency of volatile profiles across storage suggests good product stability and safety [46,85]. Optimizing date-nut ratios based on volatile profiles can therefore tailor flavor while preserving functional and healthful properties, providing a robust foundation for quality control and consumer-driven product development. The GC-MS volatile profiling analysis demonstrates that formulated Sukkari date-nut bars represent a promising category of functional food products with complex volatile profiles that contribute to both sensory appeal and potential health benefits [5]. The optimization of formulation ratios based on volatile compound profiles offers opportunities for developing customized products that meet diverse consumer preferences while maintaining nutritional integrity and food safety standards [9]. PCA and hierarchical clustering revealed that a single dominant dimension (PC1, 94.47% variance) governs compositional differences among the five date bar formulations, reflecting deliberate variation in ingredient proportions. Samples formed a transparent gradient from nutrient-dense (DNB1–DNB2) to carbohydrate-rich (DNB4–DNB5) profiles, corroborated by clustering patterns. Strong positive loadings of minerals, proteins, and antioxidant compounds versus negative loadings of moisture and carbohydrates indicate a trade-off between nutrient density and simple carbohydrates, in line with previous food formulation studies. This multivariate approach underscores its utility for guiding formulation optimization in functional food development [9,40].

In summary, higher date-to-nut ratios shift bars from protein- and fat-rich (DNB1–DNB3) toward carbohydrate-rich formulations (DNB4–DNB5), altering mineral density, antioxidant capacity, and protein quality. DNB1–DNB2 suit sports-nutrition, while DNB4–DNB5 favor general snacking. Limitations include in vitro digestibility and no sensory data; future work should assess in vivo bioavailability, consumer acceptability, and bioactive stabilization.

## 5. Conclusions

The study demonstrates that high-energy bars formulated with Sukkari date paste and mixed nuts yield distinct nutritional and functional profiles that can be tailored by adjusting date-to-nut ratios: nut-rich formulations optimize protein content, essential AA balance, mineral density, and antioxidant capacity, while date-rich variants enhance natural carbohydrate provision and palatability. A 60:40 date-to-nut ratio strikes an optimal balance, maximizing phenolic recovery, antioxidant activity, and sensory appeal, positioning such bars as versatile functional foods for athletes, health-conscious consumers, and populations with specific dietary needs. Future work should focus on clinical trials to validate metabolic and performance benefits, process optimization for industrial scale-up and shelf-life stability, sensory evaluation across demographics, and the incorporation of additional bioactives or probiotics. Sustainability assessments of ingredient sourcing and the development of targeted formulations such as low-glycemic, mineral-fortified, or age-specific bars will further enhance the commercial and public health impact of these nutritionally engineered snack products.

## Figures and Tables

**Table 1:** Formulas of high-energy date-nuts-based (DNB) bars (g 100 g^−1^).

Ingredients	DNB1	DNB2	DNB3	DNB4	DNB5
Sukkari date paste	40.00	50.00	60.00	70.00	80.00
dried Sukkari date powder	12.00	10.00	8.00	6.00	4.00
Roasted almond	9.00	7.50	6.00	4.50	3.00
Pistachio	6.00	5.00	4.00	3.00	2.00
Whole oats	6.00	5.00	4.00	3.00	2.00
Cow’s milk powder	6.00	5.00	4.00	3.00	2.00
Roasted cashew	4.50	3.75	3.00	2.25	1.50
Roasted sesame seeds	4.50	3.75	3.00	2.25	1.50
Walnuts	3.00	2.50	2.00	1.50	1.00
Hulled pumpkin seeds	3.00	2.50	2.00	1.50	1.00
Hulled sunflower seeds	3.00	2.50	2.00	1.50	1.00
Chia seeds	1.80	1.50	1.20	0.90	0.60
Dried cinnamon powder	0.60	0.50	0.40	0.30	0.20
Dried ginger powder	0.60	0.50	0.40	0.30	0.20

DNB: date-nuts-based bars.

**Table 2:** Nutritional composition of different formulated high-energy DNBs (mean ± SE), *n* = 3.

Composition *	High-Energy DNB Formulas				
	DNB1	DNB2	DNB3	DNB4	DNB5
Moisture	9.68 ± 0.14 ^a^	10.61 ± 0.16 ^b^	11.55 ± 0.15 ^c^	12.49 ± 0.21 ^d^	13.43 ± 0.18 ^e^
Protein	10.89 ± 0.16 ^a^	9.56 ± 0.15 ^b^	8.23 ± 0.11 ^c^	6.90 ± 0.11 ^d^	5.56 ± 0.07 ^e^
Total fat	18.66 ± 0.27 ^a^	15.65 ± 0.24 ^b^	12.64 ± 0.17 ^c^	9.63 ± 0.16 ^d^	6.62 ± 0.09 ^e^
Ash	3.16 ± 0.05 ^a^	3.11 ± 0.05 ^ab^	3.04 ± 0.04 ^b^	2.98 ± 0.05 ^bc^	2.92 ± 0.04 ^c^
Dietary Fiber	6.23 ± 0.09 ^a^	6.11 ± 0.09 ^a^	5.99 ± 0.08 ^a^	5.87 ± 0.1 ^a^	5.74 ± 0.08 ^a^
Total carbohydrates	57.62 ± 0.82 ^a^	61.08 ± 0.94 ^b^	64.54 ± 0.86 ^c^	68.01 ± 1.13 ^d^	71.47 ± 0.95 ^e^
Energy (kcal)	417.01 ± 5.96 ^a^	398.95 ± 5.70 ^b^	380.88 ± 5.44 ^c^	362.81 ± 5.18 ^d^	344.74 ± 4.92 ^e^

*: Presented on 100 g wet weight, SE: Standard error, ^a,b,c,d,e^: No significant difference (*p* > 0.05) between any two means within the same row with the same superscripted letters.

**Table 3:** Mineral content (mg 100 g^−1^) * of different formulated high-energy DNBs (mean ± SE), *n* = 3.

Minerals and Trace Elements (mg kg^−1^)	High-Energy DNB Formulas				
	DNB1	DNB2	DNB3	DNB4	DNB5
Calcium	422.12 ± 7.04 ^a^	360.1 ± 6.55 ^b^	324.88 ± 6.5 ^c^	296.37 ± 5.39 ^d^	241.06 ± 4.02 ^e^
Sodium	57.54 ± 0.96 ^a^	49.95 ± 0.91 ^b^	45.87 ± 0.92 ^c^	42.68 ± 0.78 ^d^	35.97 ± 0.6 ^e^
Potassium	1247.0 ± 20.78 ^a^	1165.9 ± 21.2 ^b^	1147.59 ± 22.95 ^b^	1145.02 ± 20.82 ^b^	1079.54 ± 17.99 ^c^
Phosphorus	785.95 ± 13.1 ^a^	665.46 ± 12.1 ^b^	595.68 ± 11.91 ^c^	538.58 ± 9.79 ^d^	430.77 ± 7.18 ^e^
Magnesium	326.52 ± 5.44 ^a^	281.1 ± 5.11 ^b^	256.01 ± 5.12 ^c^	235.99 ± 4.29 ^d^	195.66 ± 3.26 ^e^
Manganese	3.57 ± 0.06 ^a^	3.02 ± 0.05 ^b^	2.70 ± 0.09 ^c^	2.44 ± 0.11 ^d^	1.95 ± 0.03 ^e^
Copper	1.67 ± 0.07 ^a^	1.41 ± 0.03 ^ab^	1.26 ± 0.11 ^b^	1.14 ± 0.12 ^bc^	0.91 ± 0.08 ^c^
Iron	6.97 ± 0.12 ^a^	5.96 ± 0.11 ^b^	5.38 ± 0.11 ^c^	4.92 ± 0.09 ^d^	4.02 ± 0.07 ^e^
Zinc	5.46 ± 0.09 ^a^	4.60 ± 0.08 ^b^	4.09 ± 0.08 ^c^	3.68 ± 0.07 ^d^	2.91 ± 0.05 ^e^
Selenium (µg kg^−1^)	21.89 ± 0.36 ^a^	18.49 ± 0.34 ^b^	16.51 ± 0.33 ^c^	14.88 ± 0.27 ^d^	11.84 ± 0.21 ^e^

*: Presented on wet weight, SE: Standard error, ^a,b,c,d,e^: No significant difference (*p* > 0.05) between any two means within the same row with the same superscripted letters.

**Table 4:** Antioxidant activity, total phenolic content, total flavonoids, and total flavonols in different formulated high-energy DNBs (mean ± SE), *n* = 3.

Phytochemical Analysis *	High-Energy DNB Formulas				
	DNB1	DNB2	DNB3	DNB4	DNB5
TPC [mg GAE g^−1^]	37.98 ± 2.37 ^a^	33.77 ± 3.52 ^ab^	25.98 ± 1.35 ^bc^	22.08 ± 3.37 ^bc^	18.17 ± 2.24 ^cd^
DPPH-RSA [µmol of TE g^−1^]	48.29 ± 6.69 ^a^	40.53 ± 5.62 ^ab^	29.88 ± 3.40 ^c^	29.26 ± 2.49 ^cd^	23.09 ± 4.31 ^d^
ABTS-RSA [µmol of TE g^−1^]	62.76 ± 8.91 ^a^	48.65 ± 5.75 ^ab^	38.84 ± 6.52 ^bc^	36.57 ± 4.61 ^c^	27.71 ± 2.37 ^d^
TF [mg QE g^−1^]	26.01 ± 3.37 ^a^	20.26 ± 3.31 ^ab^	15.33 ± 2.2 ^b^	12.14 ± 1.2 ^c^	10.91 ± 0.91 ^d^
TFL [mg QE g^−1^]	20.80 ± 1.73 ^a^	15.19 ± 2.33 ^b^	9.96 ± 1.53 ^c^	9.11 ± 2.15 ^c^	7.75 ± 2.21 ^c^

*: Presented on wet weight, SE: Standard error, ^a,b,c,d^: No significant difference (*p* > 0.05) between any two means within the same column with the same superscripted letters.

**Table 5:** The phenolic acids and flavonoids content (mg kg^−1^) * in formulated DNBs.

RT	Name	DNB1	DNB3	DNB5
Phenolic acids (mg kg^−1^)				
2.97	Gallic acid	10.44	15.56	10.73
3.43	Catechol	4183.11	4640.26	3676.47
3.89	*p*-hydroxybenzoic acid	76.03	89.09	75.66
5.33	Vanillic acid	495.79	528.95	418.57
6.73	Syringic acid	0	5.31	0
6.92	*p*-coumaric	2.38	0	2.87
9.22	O-cumaric	3.92	3.22	0
Flavonoids (mg kg^−1^)				
9.64	Rutin	2.11	1.35	0
10.85	Myricetin	0.59	0.25	0
12.1	Quercetin	2.79	0.66	5.82
14.04	Kaempferol	0.14	0.39	0
14.55	Apigenin	1.48	0.69	0

* Presented means are the average of duplicate analyses.

**Table 6:** AA composition (g 100 g^−1^ bar) * of different formulated high-energy DNBs.

AAs	DNB1	DNB2	DNB3	DNB4	DNB5
Essential AAs (EAAs)					
Leucine	0.57	0.55	0.50	0.42	0.33
Lysine	0.33	0.32	0.28	0.24	0.19
Valine	0.43	0.42	0.35	0.32	0.30
Methionine	0.14	0.13	0.10	0.09	0.11
Histidine	0.35	0.32	0.28	0.26	0.22
Threonine	0.29	0.27	0.24	0.22	0.17
Phenylalanine	0.45	0.46	0.396	0.36	0.31
Isoleucine	0.25	0.25	0.21	0.18	0.13
Cystine	0.22	0.23	0.1769	0.16	0.20
Non-Essential AAs (NEAAs)					
Aspartic Acid	0.84	0.79	0.72	0.64	0.50
Glutamic Acid	1.92	1.80	1.64	1.46	1.08
Serine	0.43	0.41	0.37	0.33	0.26
Glycine	0.43	0.40	0.37	0.33	0.25
Arginine	0.64	0.71	0.62	0.46	0.34
Alanine	0.41	0.38	0.36	0.32	0.25
Tyrosine	0.08	0.12	0.87	0.07	0.09
Proline	0.69	0.43	0.56	0.42	0.56
Essential AAs	3.03	2.95	2.52	2.24	1.97
Non-Essential AAs	5.45	5.04	5.50	4.03	3.32
EAAs/NEAAs ratio	0.56	0.59	0.46	0.56	0.59
Total AAs	8.47	7.99	8.02	6.26	5.29

*: Presented means are the average of duplicate analyses.

**Table 7:** The AAs% and calculated biological efficiency, essential AA index, estimated protein efficiency ratio, and requirement index of different age groups.

Parameters	DNB1	DNB2	DNB3	DNB4	DNB5
Total BCAAs (mg g^−1^ protein)	148.07	152.71	131.33	145.75	144.80
Total BAAs (mg g^−1^ protein)	155.50	168.73	147.42	154.05	141.78
Total Aromatic AA (mg g^−1^ protein)	62.29	73.48	157.89	67.53	75.05
Total uncharged polar AAs (mg g^−1^ protein)	445.61	452.50	500.00	458.49	433.08
Total Conditional AA (mg g^−1^ protein)	469.21	462.39	526.57	462.32	475.80
BV	43.06	50.44	45.84	45.09	52.05
EAAI	54.09	56.27	53.58	53.90	55.92
Requirement index (infants)	115.89	120.55	114.79	115.47	119.80
Requirement index (preschool child)	125.86	130.93	124.67	125.41	130.11
Requirement index (schoolchild)	137.73	143.27	136.42	137.23	142.38
Requirement index (adult)	144.84	150.67	143.47	144.32	149.73

BAAs: Basic AAs, BV: Calculated biological value, EAAI: Essential AA index.

**Table 8:** FA composition (g 100 g^−1^) * of high-energy DNBs.

	DNB1	DNB3	DNB5
Saturated FAs	%	%	%
Butyric (C4:0)	0.19	0.24	0.32
Caproic (C6:0)	0.14	0.18	0.28
Caprylic (C8:0)	0.09	0.15	0.19
Capric (C10:0)	0.24	0.29	0.57
Lauric (C12:0)	0.39	0.53	0.77
Myristic (C14:0)	1.85	2.18	3.16
Myristoleic (C14:0)	0.06	0.21	0.11
Pentadecanoic (C15:0)	0.22	0.26	0.38
Palmitic (C16:0)	15.06	16.39	20.26
Margaric acid (C17:0)	0.17	0.19	0.24
Stearic (C18:0)	5.33	5.44	6.42
Arachidic (C20:0)	0.24	0.22	0.22
Behenic (C22:0)	0.13	0.12	0.11
Monounsaturated FAs			
cis-10-pentadecenoic acid (C15:1)	0.03	0.04	0.06
Palmitoleic (C16:1)	0.51	0.62	0.62
cis-10-Heptadecenoic acid (C17:1)	0.09	0.08	0.08
Oleic (C18:1 n9)	44.94	43.76	40.51
Polyunsaturated FAs			
Arachidonic acid (20:4 n6)	0.03	0.04	0.06
Linoleic (C18:2 n6)	27.38	26.12	23.12
Linolenic (C18:3 n3)	2.9	2.92	2.52

*: Presented means are the average of duplicate analyses.

**Table 9:** GC-MS volatile profile analysis of different formulated DNBs.

Name	RT	DNB1	DNB3	DNB5
Benzene, (1-butylhexyl)-	18.06	1.68	1.13	1.71
Benzene, (1-propylheptyl)-	18.26	1.38	0.91	1.45
Benzene, (1-ethyloctyl)-	18.68	1.16	0.74	1.23
Benzene, (1-methylnonyl)-	19.52	1.05	-	1.08
Benzene, (1-pentylhexyl)-	20.24	1.67	0.99	1.82
Benzene, (1-butylheptyl)-	20.31	4.31	2.53	4.63
Benzene, (1-propyloctyl)-	20.53	3.1	1.82	3.4
Benzene, (1-ethylnonyl)-	20.98	2.59	1.51	2.76
Benzene, (1-methyldecyl)-	21.79	2.46	1.54	2.55
Benzene, (1-pentylheptyl)-	22.38	4.65	3.82	4.77
Benzene, (1-butyloctyl)-	22.47	4.95	3.15	5.37
Benzene, (1-propylnonyl)-	22.73	3.44	2.08	3.65
Benzene, (1-ethyldecyl)-	23.17	2.72	1.58	2.92
Benzene, (1-methylundecyl)-	23.97	2.39	1.39	2.78
Benzene, (1-pentyloctyl)-	24.44	3.74	2.29	4.01
Benzene, (1-butylnonyl)-	24.57	2.92	1.61	2.99
Benzene, (1-propylheptadecyl)-	24.83	2.18	1.06	2
Benzene, (1-ethylundecyl)-	25.29	1.78	0.85	1.56
7-Hexadecenoic acid, methyl ester, (Z)-	25.88	0.00	0.48	0.00
Benzene, (1-methyldodecyl)-	26.06	1.73	1.07	1.68
Hexadecanoic acid, methyl ester	26.4	7.74	12.6	9.07
17-Octadecynoic acid	27.63	-	-	0.38
9,12-Octadecadienoic acid (Z, Z)-, methyl ester	29.42	12.8	16.06	6.65
9-Octadecenoic acid, methyl ester,(E)-	29.59	25.31	30.08	19.21
Methyl stearate	30.15	3.29	6.52	4.97
Hexadecanoic acid, 2-hydroxy-1-(hydroxymethyl)ethyl ester	32.08	-	0.62	0.72
9-Octadecenoic acid (Z)-	32.19	-	-	1.08
9 12-octadecadienoic acid (Z)- 2-hydroxy-1-(hydroxymethyl)ethyl ester	34.78	-	0.59	0.58
9,12-Octadecadienoyl chloride, (Z, Z)-	34.94	0.84	2.99	3.59
Mono(2-ethylhexyl) phthalate	36.62	0.38	-	1
1,2-Benzenedicarboxylic acid	39.81	-	-	0.14

- Not detected.

## Data Availability

The original contributions presented in this study are included in the article. Further inquiries can be directed to the corresponding author.
